# Factors determining the oral absorption and systemic disposition of zeaxanthin in rats: *in vitro*, *in situ*, and *in vivo* evaluations

**DOI:** 10.1080/13880209.2022.2143534

**Published:** 2022-11-22

**Authors:** Seong‑Wook Seo, Dong‑Gyun Han, Eugene Choi, Min‑Jeong Seo, Im‑Sook Song, In‑Soo Yoon

**Affiliations:** a Department of Manufacturing Pharmacy, College of Pharmacy and Research Institute for Drug Development, Pusan National University, Busan, South Korea; b Freshwater Biosources Utilization Bureau, Bioresources Industrialization Support Division, Nakdong‑gang National Institute of Biological Resources (NNIBR), Sangju‑si, South Korea; c BK21 FOUR Community‑Based Intelligent Novel Drug Discovery Education Unit, Vessel‑Organ Interaction Research Center (VOICE), Research Institute of Pharmaceutical Sciences, College of Pharmacy, Kyungpook National University, Daegu, South Korea

**Keywords:** Physicochemical property, gut absorption, tissue distribution, hepatic clearance, lymphatic absorption, oral bioavailability

## Abstract

**Context:**

Zeaxanthin is a yellow‑coloured dietary carotenoid widely recognized as an essential component of the macula. It exerts blue light filtering and antioxidant activities, offering eye health and vision benefits.

**Objective:**

This study explores the oral absorption and systemic disposition of zeaxanthin from biopharmaceutical and pharmacokinetic perspectives.

**Materials and methods:**

*In vivo* intravenous (5 and 10 mg/kg) and intraportal (5 mg/kg) pharmacokinetic studies were performed to determine intrinsic tissue‑blood partition coefficient, elimination pathway, and hepatic clearance, of zeaxanthin in rats. Moreover, *in vitro* physicochemical property test, *in situ* closed loop study, *in vivo* oral pharmacokinetic study (20 and 100 mg/kg), and *in vivo* lymphatic absorption study (100 mg/kg) were conducted to investigate the gut absorption properties of zeaxanthin and assess the effects of several lipids on the lymphatic absorption of zeaxanthin in rats.

**Results:**

Zeaxanthin exhibited poor solubility (≤144 ng/mL) and stability (6.0–76.9% of the initial amount remained at 24 h) in simulated gut luminal fluids. Gut absorption of zeaxanthin occurred primarily in the duodenum, but the major fraction (≥84.7%) of the dose remained unabsorbed across the entire gut tract. Considerable fractions of intravenous zeaxanthin accumulated in the liver, lung, and spleen (21.3, 11.7, and 2.0%, respectively). It was found that the liver is the major eliminating organ of zeaxanthin, accounting for 53.5–90.1% of the total clearance process (hepatic extraction ratio of 0.623).

**Discussion and conclusions:**

To our knowledge, this is the first systematic study to report factors that determine the oral bioavailability and systemic clearance of zeaxanthin.

## Introduction

**Figure 1.**
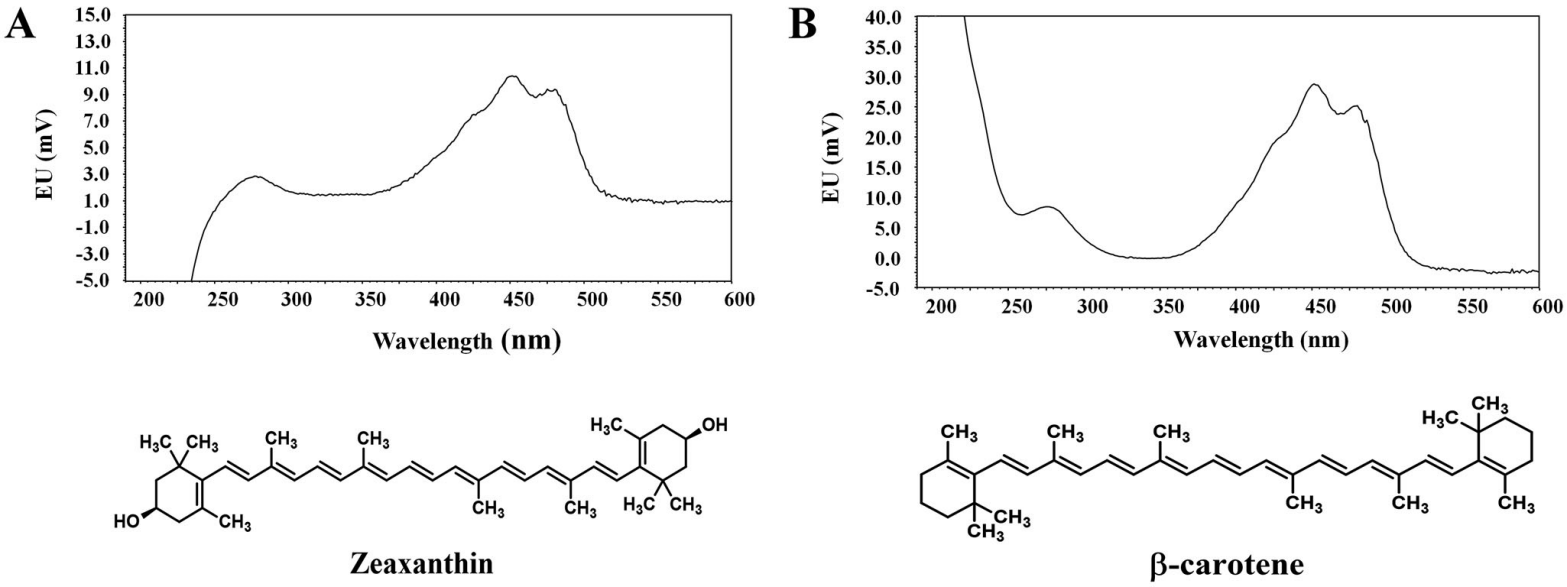
Chemical structures and ultraviolet-visible (UV-Vis) absorbance spectra of (A) zeaxanthin and (B) IS (dissolved 1000 ng/mL in methanol solution).

Carotenoids are a class of naturally occurring yellow, orange, and red pigments, synthesized *de novo* by photosynthetic plants, algae, bacteria, and fungi (Maoka 2020). Generally, α‑carotene, β‑carotene, β‑cryptoxanthin, lutein, lycopene, and zeaxanthin (Figure 1) are the major dietary carotenoids prevalent in human serum and tissues (Mein et al. 2011; Bernstein et al. 2016; Toti et al. 2018). Carotenoids can be structurally categorized into two classes: carotenes and xanthophylls. Carotenes, such as α‑carotene, β‑carotene, and lycopene, are non‑polar compounds that are pure hydrocarbons without oxygen atoms (Jia et al. 2017). Xanthophylls, such as lutein, zeaxanthin, and β‑cryptoxanthin, are relatively polar carotenoids that contain at least one oxygen atom (Jia et al. 2017). α‑Carotene, β‑carotene, and β‑cryptoxanthin are provitamin A carotenoids that can be metabolized to retinol after administration in the body, whereas lutein, zeaxanthin, and lycopene are non‑provitamin A carotenoids that cannot be metabolized to retinol (Mein et al. 2011).

Zeaxanthin (β,β‑carotene‑3,3′‑diol) is a yellow‑coloured tetraterpene pigment with a molecular weight of 568.8 Da (Murillo et al. 2019). Zeaxanthin, along with lutein and *meso*‑zeaxanthin, is highly concentrated in the macula of the retina that is mainly responsible for central and fine‑feature vision (Billsten et al. 2003; Eisenhauer et al. 2017). These three carotenoids (called macular pigments) are efficient absorbers of blue light, protecting against age‑related macular degeneration (AMD), a degenerative disease that may lead to blurred or no vision in the centre of the visual field, owing to their blue light‑filtering and antioxidant activities (Kijlstra et al. 2012; Mares 2016). Dietary intake and plasma levels of these carotenoids have been associated with a lower risk of AMD (Hartmann et al. 2004). Additionally, zeaxanthin acts as a more potent antioxidant than lutein, protecting against oxidative stress in other tissues as well as the eyes (Murillo et al. 2019). Mammals are not able to synthesize zeaxanthin; thus, it must be obtained from dietary sources (Delgado‑Pelayo and Hornero‑Mendez 2012). Zeaxanthin is found in many plants, including green leafy and yellow‑orange vegetables and fruits, such as carrots, corn, orange, paprika, saffron, and wolfberries (goji) (Murillo et al. 2019). It can also be found in animal products, such as egg yolks and cheese (Murillo et al. 2019).

Numerous researches have demonstrated that oral doses of phytochemicals often cannot produce sufficient blood concentrations to exert therapeutic efficacy in clinical settings, despite their health benefits (Han et al. 2021). If this holds true for zeaxanthin, systemic investigations from biopharmaceutical and pharmacokinetic perspectives could be useful for the rational design of efficient oral formulations and dosing regimens for zeaxanthin. Following uptake by enterocytes mainly *via* passive diffusion, zeaxanthin is packed into chylomicrons and released into the lymph, or it is directly incorporated into the intestine‑derived high‑density lipoprotein (HDL) and secreted into the portal vein or the lymph (Deming and Erdman 1999; Niesor et al. 2014; Murillo et al. 2019). It has been reported that zeaxanthin is found predominantly in HDL (53%), followed by low‑density and very low‑density lipoproteins, in the systemic circulation (Blesso et al. 2013). It is generally thought that carotenoids are distributed primarily into the liver and adipose tissues and are also found in the adrenal glands, kidneys, and testes (Kiokias et al. 2016). In mammals, two enzymes, carotene‑15,15′‑monooxygenase (CMO1) and carotene‑9′,10′‑monooxygenase (CMO2), are primarily responsible for the metabolism of carotenoids (Giordano and Quadro 2018). CMO2 catalyzes the exocentric cleavage of xanthophylls, including zeaxanthin, whereas CMO1 is not involved in the metabolism of zeaxanthin (Redmond et al. 2001; Mein et al. 2011; Giordano and Quadro 2018).

There have been a few studies that reported zeaxanthin pharmacokinetics. Hartmann et al. (2004) reported that long‑term oral intake of 1 and 10 mg zeaxanthin as beadlets increased plasma zeaxanthin concentrations in healthy volunteers. Edwards reviewed the existing results of mass balance and distribution studies in rats fed orally with zeaxanthin‑enriched diet and liposomal preparation of (^14^C)‑zeaxanthin (Edwards 2016). However, these previous studies just focussed on the concentration profiles of zeaxanthin in plasma, tissues, and excreta after multiple oral intakes (with undefined doses in some cases) in a descriptive manner, which is not sufficient to properly address the biopharmaceutic and pharmacokinetic characteristics of zeaxanthin. To the best of my knowledge, there have been no comprehensive studies to elucidate kinetic factors determining the oral absorption and systemic disposition of zeaxanthin through more quantitative and mechanistic approaches.

Therefore, in the present study, *in vivo* intravenous and intraportal pharmacokinetic studies were conducted to investigate its systemic disposition properties, such as intrinsic tissue‑blood partition coefficient, elimination pathway, and hepatic clearance, of zeaxanthin in rats. Moreover, *in vitro* physicochemical property test, *in situ* closed loop study, *in vivo* oral pharmacokinetic study, and *in vivo* lymphatic absorption study were conducted to investigate the gut absorption properties of zeaxanthin and assess the effects of several lipids on the lymphatic absorption of zeaxanthin in rats. The results were comprehensively analyzed based on the concepts of mass balance and well‑stirred organ clearance. The present study is expected to offer significant insights into the development of dosage regimens and oral formulation of zeaxanthin.

## Materials and methods

### Materials and animals

Zeaxanthin (purity > 95%) and β‑carotene [purity ≥ 98%; used as an internal standard (IS)] were purchased from Biosynth Carbosynth (Newbury, UK). HPLC‑grade acetonitrile, methanol, dimethyl sulfoxide, and methyl *t*‑butyl ether (MTBE) were purchased from Honeywell, Inc. (Muskegon, MA, USA). Simulated gastric fluid (SGF), with pH 1.2, was purchased from Wako Pure Chemical Co. (Osaka, Japan). Buffer solutions (pH 1.0, 3.0, 5.0, 7.0, 9.0, and 11.0) and simulated intestinal fluid (SIF), with pH 6.8, were purchased from Samchun Pure Chemical Co., Ltd. (Gyeonggi‑do, South Korea). Pooled human blood and plasma were purchased from Innovative Research, Inc. (Novi, MI, USA). Eight‑week‑old male Sprague–Dawley rats (240–280 g) were purchased from Koatech Co., Ltd. (Gyeonggi‑do, South Korea). Rats were individually housed in clean cages at an ambient temperature of 23 °C. The animal study protocols were approved by the Institutional Animal Care and Use Committee of the Pusan National University for scientific care and ethical procedures (approval number: PNU‑2021‑2949).

### Bioanalytical procedures and method validation

The zeaxanthin concentration in the samples obtained from the experiments conducted in this study was determined using a validated HPLC method. A Shimadzu HPLC system coupled with an ultraviolet‑visible (UV‑Vis) detector (SPD‑20A; Shimadzu Co., Kyoto, Japan) was used in this study. The light wavelengths that produced the maximum UV‑Vis absorbance intensity were chosen based on the UV‑Vis absorbance spectrum measurement for zeaxanthin and the IS (1000 ng/mL in methanol solution). Chromatographic separation was performed at 30 °C using a YMC Carotenoid C30 column (250 × 4.6 mm, 5 μm, 100 Å; YMC KOREA Co., Ltd., Gyeonggi‑do, South Korea) connected to a C18 guard column (Security Guard HPLC Cartridge System, Phenomenex). Gradient elution of the mobile phase consisting of deionized water (DW; solvent A), methanol (solvent B), and MTBE (solvent C) was performed at a flow rate of 1 mL/min, and the procedure was as follows (solvent A:solvent B:solvent C, v/v/v): started at 10:60:30 at 0 min, ramped from 10:60:30 to 10:20:70 for 0.1 min, maintained at 10:20:70 for 5.5 min, back to 10:60:30 for 0.1 min, and maintained for 6.5 min (hence, the total run time was 20 min). plasma samples (120 μL) were deproteinized with 120 μL of ice‑cold methanol/MTBE mixture (1:1, v/v) containing IS (2000 ng/mL) and 600 μL of ethyl acetate. The resultant mixture was vortexed for 10 min and centrifuged at 16,000 × *g* for 10 min. Next, 500 μL of the resultant supernatant was transferred to a clean microtube and evaporated using a SpeedVac centrifugation system (CVE‑3110; EYELA Co., Tokyo, Japan). The resultant dried residue was reconstituted with 60 μL of a methanol/MTBE/ethyl acetate mixture (1:1:1, v/v/v), and a 30 μL aliquot was injected into the HPLC system.

The bioanalytical method proposed herein was validated by analyzing calibration standards and quality control (QC) samples according to US FDA guidelines (USA Food and Drug Administration 2018). Stock solutions of zeaxanthin and IS were prepared at 1000 μg/mL in dimethyl sulfoxide. A stock solution of zeaxanthin was serially diluted with methanol to prepare working standard solutions with final concentrations of 0.01–10 μg/mL. To prepare the calibration standards, blank rat plasma was spiked with each working standard solution to obtain final concentrations of 10, 20, 50, 100, 200, 500, 1000, 2000, 5000, and 10,000 ng/mL. The QC samples were prepared using the same process as that used for the calibration standards. The QC sample levels were set as follows: 10 (LLOQ; lower limit of quantification), 30 (LQC; low QC level), 250 (MQC; middle QC level), and 8000 ng/mL (HQC; high QC level). The linearity, sensitivity, selectivity, precision, accuracy, extraction recovery, and matrix effects of the method were assessed as previously described (Seo et al. 2021, 2022). Analytical stability was determined under various handling and storage conditions, such as benchtop, freeze‑thaw, post‑preparative, and long‑term storage, at two different QC levels (LQC and HQC).

### *In vitro* determination of physicochemical properties of zeaxanthin

The lipophilicity, solubility, plasma protein binding, blood distribution, and biological stability of zeaxanthin were determined as described in our previous study (Han et al. 2021). The distribution coefficient (log *D*) was measured in buffers of varying pH values (pH 1.0, 3.0, 5.0, 7.0, 9.0, and 11.0). The SGF and SIF used in the solubility and stability tests were prepared as follows: SGF was prepared by dissolving 0.32% (w/v) pepsin, 0.2% (w/v) sodium chloride, and 0.7% (v/v) HCl in DW (final pH = 1.2); SIF was prepared by dissolving 0.1% (w/v) pancreatin and 3 mM sodium taurocholate in phosphate buffer (final pH = 7.0). The solubility of zeaxanthin (2 μM) was measured in buffers of varying pH values (pH 1.0, 3.0, 5.0, 7.0, 9.0, and 11.0), plasma, phosphate buffer saline, SGF, and SIF. The unbound fraction in plasma (fu_P_) and the blood‑to‑plasma concentration ratio (*R_B_*) were determined at a zeaxanthin concentration of 2 μM. The stability of zeaxanthin (2 μM) was determined in several matrices, including buffers of varying pH values (pH 1.0, 3.0, 5.0, 7.0, 9.0, and 11.0), plasma, urine, SGF, and SIF.

### *In vivo* pharmacokinetic study in rats

Rats underwent surgery for cannula implantation into the femoral vein and artery under light ether anaesthesia (Kim et al. 2019; Park et al. 2021). Zeaxanthin was dissolved in a vehicle solution consisting of dimethyl sulfoxide, ethanol, and polyethylene glycol 400 (20:5:75, v/v/v) for intravenous dosing (5 and 10 mg/kg), and a vehicle solution consisting of corn oil, polyethylene glycol 400, propylene glycol, Tween 80, and saline (10:20:10:30:30, v/v/v/v/v) was used for oral dosing (20 and 100 mg/kg). Blood samples (250 μL) were collected in heparin‑coated microcentrifuge tubes *via* the femoral artery at 0, 2, 5, 15, 30, 60, 90, 120, 180, 240, and 360 min following intravenous administration and at 0, 5, 15, 30, 60, 90, 120, 180, 240, 360, 480, 1200, and 1440 min following oral administration. The blood sample was centrifuged at 3000 × *g* for 10 min at 4 °C, and 100 μL of the resultant supernatant (plasma sample) was collected. Urine was collected in a 15 mL tube over a period of 24 h, in parallel with blood collection. Subsequently, the rats were euthanized by carbon dioxide inhalation, and the entire gut tract, including its contents and faeces, was removed, transferred into a beaker, and cut into pieces using scissors. Next, 200 mL of a methanol/MTBE mixture (1:1, v/v) was added to the beaker, followed by gentle stirring with a glass rod for 1 min, and 120 μL of the resultant supernatant was collected. Plasma, urine, and gut luminal extract samples were analyzed using the validated HPLC method.

The femoral vein, femoral artery, and portal vein were cannulated as described in our previous studies (Yoon et al. 2011; Seo et al. 2022). For intravenous administration, zeaxanthin (5 mg/kg) was infused over 30 min into the femoral vein, and simultaneously, the same volume of vehicle solution consisting of dimethyl sulfoxide, ethanol, and polyethylene glycol 400 (20:5:75, v/v/v) was infused over 30 min into the portal vein (infusion pump: LEGATO^®^ 101 syringe pump, KD Scientific Inc., MA, USA). For intraportal administration, the same dose of zeaxanthin was infused over 30 min into the portal vein, and simultaneously, the same volume of the vehicle solution was infused over 30 min into the femoral vein. Approximately 250 μL of blood was collected *via* the femoral artery at 0, 15, 30, 32, 35, 45, 60, 90, 120, 150, 210, 270, 390, and 510 min following intravenous and intraportal administration. After centrifugation of the blood sample at 3000 × *g* at 4 °C for 5 min, 120 μL of plasma was obtained.

### *In situ* gut absorption in rats

After a minimal abdominal incision under light ether anaesthesia followed by sufficient washing of the contents within the gut tract, the gastric, duodenal, jejunal, ileal, and colonic closed‑loops were prepared as previously described in our previous study (Han et al. 2021). Zeaxanthin suspension (final concentration: 1 mg/mL) was prepared by dispersing zeaxanthin powder in the same vehicle solution used in the *in vivo* oral pharmacokinetic study. After injecting 0.2 mL of the zeaxanthin suspension into each loop using an l mL, 31‑gauge syringe, the entire gut tract was carefully returned to its original position in the abdominal cavity. Four hours after injection, each loop was removed, transferred into a beaker containing 40 mL of methanol, and handled similarly to the gut luminal extract samples used in the *in vivo* pharmacokinetic study. As a control group (i.e., the ‘*in vitro* isolated loop’), the loops were prepared in the same manner as the ‘*in situ* loop’ study. The loops were removed from the rat gut tract by carefully cutting with scissors, and transferred to a glass beaker. The zeaxanthin suspension was injected into the *in vitro* isolated loop. After 0 and 4 h of incubation at 37 °C, the remaining fraction of zeaxanthin in the *in vitro* isolated loop was measured in the same manner as for the gut luminal extract samples.

### *In vivo* tissue distribution study

Zeaxanthin (5 mg/kg) was intravenously injected into the femoral vein of rats. The brain, liver, heart, lung, kidney, gut, spleen, muscle, adipose, and eyes were excised 90 min after the administration, rinsed with saline, and blotted dry with clean paper. Each weighed tissue sample was homogenized (FastPrep‑24™ 5 G bead beating system, MP Biomedicals, OH, USA) in four volumes of ice‑cold saline/methanol mixture (1:1, v/v). The tissue homogenate was then centrifuged at 9000 × *g* for 10 min, and 100 μL of supernatant was collected and processed using the same procedure as that used for the plasma samples.

### *In vivo* lymphatic absorption study

Zeaxanthin was dispersed in four different vehicle systems consisting of oil phase (i.e., corn oil, stearic acid, oleic acid, or linoleic acid, respectively), polyethylene glycol 400, propylene glycol, Tween 80, and saline (10:20:10:30:30, v/v/v/v/v). Following oral administration of zeaxanthin (100 mg/kg) in rats, blood samples (250 μL) were collected in heparin‑coated microcentrifuge tubes *via* the femoral artery at 0, 5, 15, 30, 60, 90, and 120 min. The liver and mesenteric lymph tissues were excised immediately after the last blood sampling and then processed using the same procedure as described in the *in vivo* tissue distribution study.

### Data analysis

Non‑compartmental analysis was performed using WinNonlin^®^ software (Ver. 3.1, NCA200 and 201; Certara, Inc., Princeton, NJ, USA) to estimate the following pharmacokinetic parameters: total area under the plasma concentration *vs.* time curve from time zero to infinity (area under the curve; AUC), total body clearance (CL; calculated as dose/AUC), terminal half‑life (*t*_1/2_), and apparent volume of distribution at steady state (*V*_ss_). Assuming dose‑linear conditions, *F*_oral_ was calculated as the ratio of the dose‑normalized AUC between intravenous and oral administration. The peak plasma concentration (*C*_max_) and time to reach *C*_max_ (*T*_max_) were obtained directly from the experimental data. The statistical *p*‑values were estimated using the unpaired *t*‑test for comparison between two means or one‑way analysis of variance with *post-hoc* Tukey’s honestly significant difference test for comparison among three or more means; *p* < 0.05 was considered statistically significant. Unless otherwise indicated, all numerical data were rounded to three significant figures.

## Results

### Bioanalytical method development and optimization

Figure 1 shows the UV‑Vis light absorbance spectra of zeaxanthin and IS. Zeaxanthin exhibited maximum light absorbance at ∼450 nm. A YMC carotenoid C30 column (YMC KOREA Co., Ltd.) was employed as the stationary phase of the present method, as it has been recognized as one of the most representative and effective HPLC columns for carotenoid analysis. The mobile phase containing MTBE was selected based on previous studies on the bioanalytical analysis of various carotenoids, including zeaxanthin (Stinco et al. 2019; Boulet et al. 2020), and its gradient elution was fine‑tuned when other factors, such as sample pre-treatment procedures and IS, were optimized. Several carotenoids, such as β‑carotene, β‑cryptoxanthin, lutein, and lycopene, were screened for their potential use as IS. Among these, the β‑carotene peaks were best separated from zeaxanthin and other endogenous plasma component peaks, exhibiting sufficient light absorbance at the same wavelength as that used for the detection of zeaxanthin. The solvent precipitation‑reconstitution method was used to pre-treat the biological samples. Various organic solvents, such as acetonitrile, methanol, MTBE, ethyl acetate, and their mixtures, were tested. As a result, the methanol/MTBE/ethyl acetate mixture provided a low matrix effect and sufficient zeaxanthin recovery.

### Bioanalytical method validation

**Figure 2.**
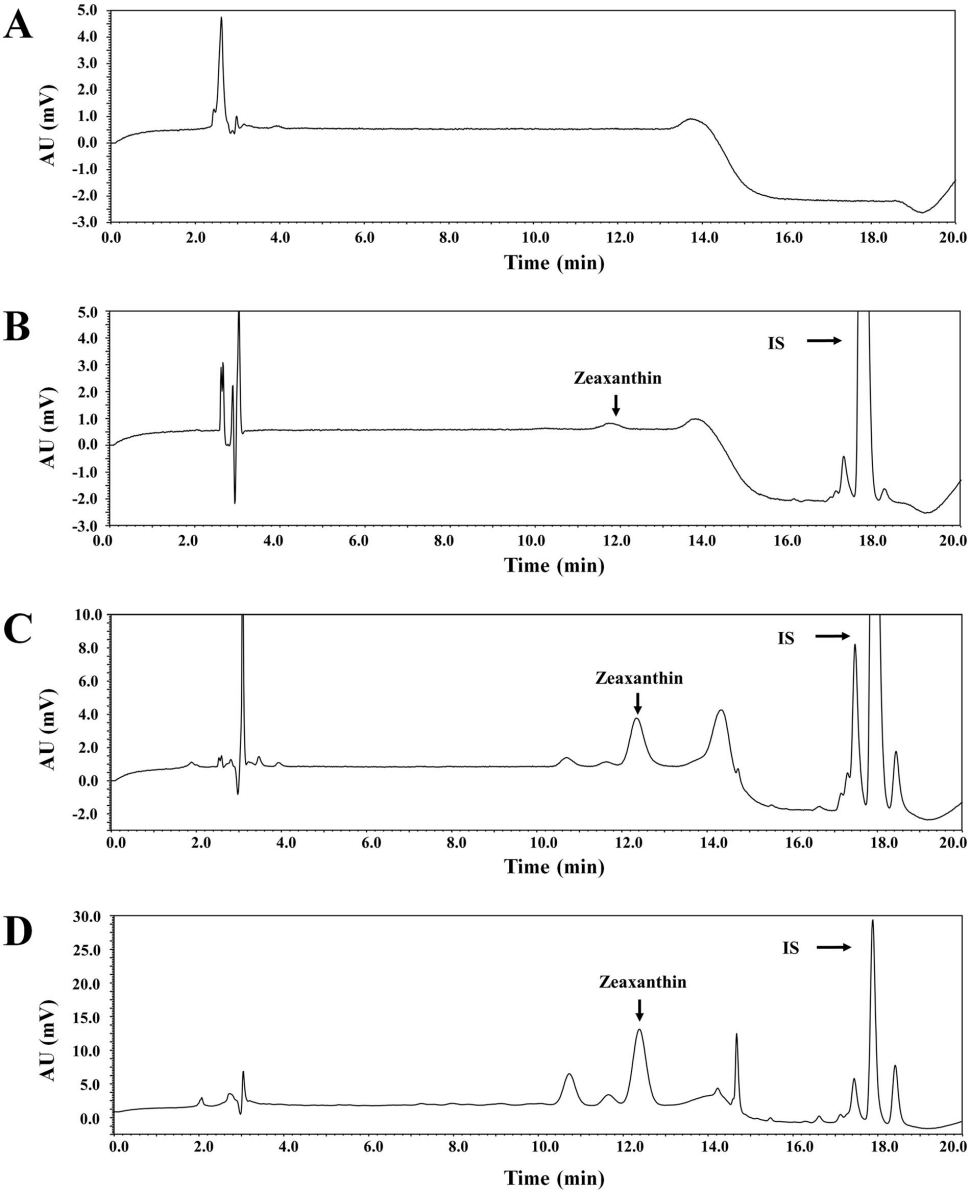
Representative chromatograms of zeaxanthin and IS in rat plasma samples: (A) blank rat plasma; (B) blank rat plasma spiked with the analytes at the LLOQ (10 ng/mL); (C) blank rat plasma spiked with the analytes at the MQC (250 ng/mL); (D) plasma sample collected at 30 min following the intravenous administration of zeaxanthin at a dose of 10 mg/kg in rats, where the calculated concentration of zeaxanthin was 1464 ng/mL.

Figure 2 shows representative chromatograms of zeaxanthin and IS in different plasma samples, thereby indicating that the analyte peaks were clearly separated from interference peaks of endogenous components present in blank plasma. The calibration curves for zeaxanthin were linear at the zeaxanthin concentration range from 10 to 10,000 ng/mL (LLOQ = 10 ng/mL). A representative equation for the calibration curve is as follows: *y* = 0.0002 × *x* + 0.0003; where *x* is the nominal concentration ratio of zeaxanthin to IS, and *y* is the peak area ratio of zeaxanthin to IS. The correlation coefficients for the calibration curves were ≥0.9993. The validation parameters of the present bioanalytical method are listed in Table 1. The within‑ and between‑run precision and accuracy of the present method were determined at four different quality control levels, namely LLOQ, LQC, MQC, and HQC; the precision was ≤7.53%, while the accuracy ranged from 92.4 to 110%. The recovery and matrix effects of the present method were determined at three quality control levels, namely LQC, MQC, and HQC; the mean recovery of zeaxanthin ranged from 85.8 to 103%, with a coefficient of variation (CV) value of ≤5.48%; the mean matrix effect of zeaxanthin ranged from 94.3 to 108%, with a CV value of ≤5.69%. The two parameters did not significantly differ among the QC levels tested (*p* = 0.718 and 0.522 for the recovery and matrix effect, respectively). Under routine experimental conditions relevant to the present bioanalytical procedures, such as being kept on the bench top and in the autosampler, frozen for storage and thawed for analysis, and stored for a relatively long period, the stability of zeaxanthin in rat plasma sample was determined at two quality control levels, namely LQC and HQC. The bias in the observed concentrations was within ±15% of the corresponding nominal values; the mean remaining fractions of zeaxanthin ranged from 89.6 to 112%, with a CV value of ≤9.14%.

**Table 1.** Within-run and between-run precision and accuracy of the present bioanalytical method for the quantitation of zeaxanthin in rat plasma (*n* = 5).

	Nominal concentration (ng/mL)			
	LLOQ (10)	LQC (30)	MQC (250)	HQC (8000)
Precision (%)				
Within-run	6.31	5.78	3.13	3.99
Between-run	3.64	2.31	1.31	3.17
Accuracy (%)				
Within-run	106 ± 6	103 ± 6	100 ± 3	92.4 ± 3.7
Between-run	104 ± 3	101 ± 2	99.6 ± 1.3	97.7 ± 3.1

### Physicochemical properties of zeaxanthin

**Figure 3.**
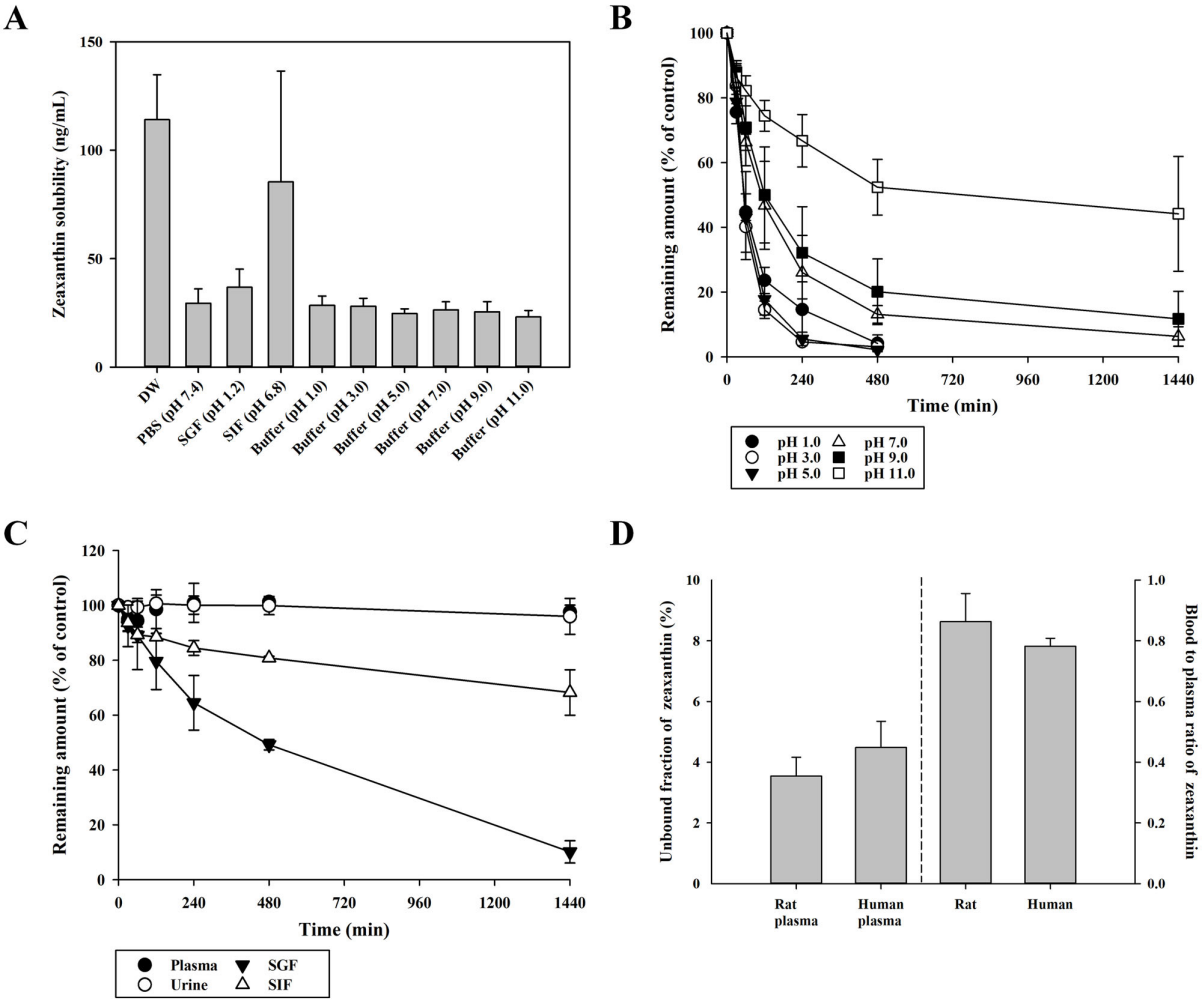
(A) Solubility in biological samples (SGF and SIF) and buffers of different pH values (pH 1, 3, 5, 7, 9, and 11), (B) stability of zeaxanthin in the buffers of different pH values and (C) biological samples over a period of 24 h, and (D) unbound fraction of zeaxanthin in rat and human plasma (in the left side of the centre dotted line) and blood-to-plasma concentration ratio (in the right side of the centre dotted line) of zeaxanthin in rat and human whole blood. The rectangular bars and circles represent the means, and the error bars represent the standard deviations (*n* = 5).

The physicochemical properties of zeaxanthin, such as lipophilicity, solubility, stability, protein binding, and blood distribution, were experimentally determined. It was difficult to determine the distribution coefficients of zeaxanthin between octanol and aqueous buffers with different pH values because its concentrations in the aqueous buffers were below the LLOQ (10 ng/mL). This indicates that zeaxanthin is a highly lipophilic compound with log *P* (or log *D*) > 4.51, which coincides with the clog *p*-values estimated using ALOGPS 2.1 and ACD software v11.02 (8.3–10.9). Figure 3 shows the equilibrium solubility and stability of zeaxanthin in the biological matrix, such as plasma, urine, SGF, and SIF, and in the buffer of varying pH values. As shown in Figure 3(A), the solubility of zeaxanthin was very low in the biological samples tested and was not dependent on the pH values. In this study, a tested compound was regarded as stable when ≥85% of the initially spiked amount remained in the sample (USA Food and Drug Administration 2018). As shown in Figure 3(B), zeaxanthin was stable in plasma and urine for 24 h and in SIF for 8 h, whereas it was unstable in SGF, that is, only 6.01–14.1% of unchanged zeaxanthin remained at the end of the incubation time. As shown in Figure 3(C), the stability of zeaxanthin tended to decrease as the pH of the buffer increased in the pH range of 1–11. In both rat and human blood, fu_P_ of zeaxanthin was very low, and *R_B_* of zeaxanthin was close to 1 (Figure 3(D)). This indicates that zeaxanthin exhibits extensive plasma protein binding and is equally distributed between red blood cells and plasma.

### Plasma pharmacokinetics of zeaxanthin in rats

**Figure 4.**
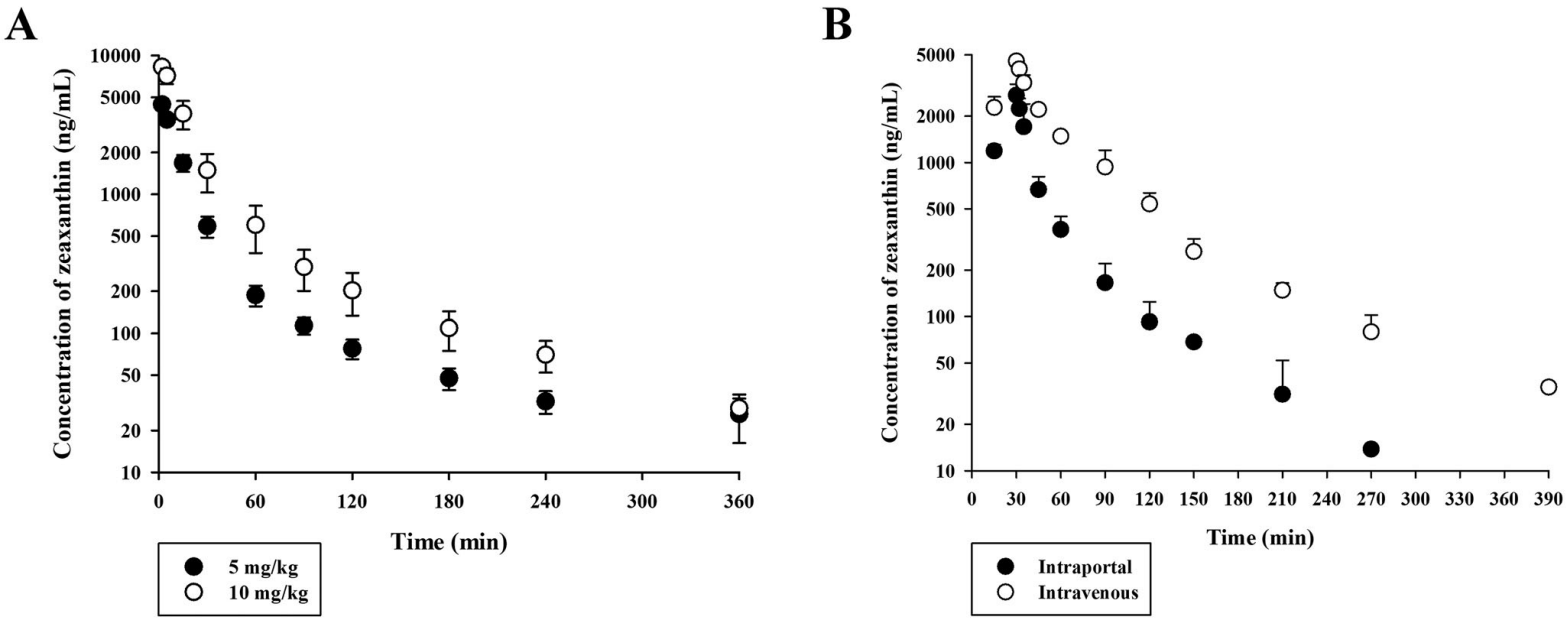
(A) Arterial plasma concentration *vs.* time profiles of zeaxanthin following intravenous administration at 5 and 10 mg/kg in rats. (B) Arterial plasma concentration *vs.* time profiles of zeaxanthin following intravenous and intraportal administration at a dose of 5 mg/kg in rats. The circles and error bars represent means and standard deviations, respectively (*n* = 5).

Figure 4(A) shows the plasma concentration *vs.* time profiles of zeaxanthin following intravenous administration at 5 and 10 mg/kg in rats, and the relevant parameters are listed in Table 2. The plasma concentrations of zeaxanthin declined in a multi‑exponential manner with *t*_1/2_ of 71.4–102 min, and relatively high *V*_ss_ and CL were observed, indicating an extensive tissue distribution and rapid elimination. The *A*_U24h_ and *A*_G24h_ values were below the detection limit, indicating negligible urinary and gut (including biliary) excretion of zeaxanthin. Notably, there were no significant differences in the pharmacokinetic parameters between the two tested doses (*p* = 0.155 for dose‑normalized AUC, 0.184 for *t*_1/2_, 0.122 for CL, and 0.740 for *V*_ss_). However, following oral administration at 50 and 100 mg/kg, the concentrations of zeaxanthin in the plasma and urine samples were below the quantitation limit, indicating extremely low oral bioavailability. Notably, considerable *A*_G24h_ values of 16.3–54.8% were observed in rats, indicating variable and limited absorption of zeaxanthin from the gut. The hepatic extraction ratio (*E_H_*) of a drug can be estimated by comparing the AUC observed after intravenous and intraportal administration. Figure 4(B) shows the plasma concentration *vs.* time profiles of zeaxanthin in rats, following intravenous and intraportal infusion over a period of 30 min at a dose of 5 mg/kg. The AUC of zeaxanthin was 243 ± 13 and 91.6 ± 14.2 μg·min/mL, following intravenous and intraportal infusion, respectively. This indicates that zeaxanthin was moderately extracted by the liver, with an *E_H_* of 0.623 [=1 − (91.6/243)].

**Table 2.** Recovery, matrix effect, and stability of zeaxanthin in rat plasma of the present bioanalytical method for quantitation of zeaxanthin in rat plasma (*n* = 5).

	Nominal concentration (ng/mL)		
	LQC (30)	MQC (250)	HQC (8000)
Recovery (%)	90.9 ± 4.8	94.4 ± 8.4	86.7 ± 2.1
Matrix effect (%)	104 ± 4	98.0 ± 9.2	102 ± 2
Stability			
Bench-top^a^	99.9 ± 6.4		108 ± 3
Autosampler^b^	106 ± 9		99.8 ± 1.4
Freeze-thaw^c^	97.1 ± 8.3		102 ± 3
Lung-term^d^	98.4 ± 5.3		102 ± 2

^a^Room temperature for 3 h.

^b^10 °C for 24 h in the autosampler.

^c^Three freezing and thawing cycles.

^d^−20 °C for 30 day.

### Gut absorption, tissue distribution, and lymphatic absorption of zeaxanthin in rats

**Figure 5.**
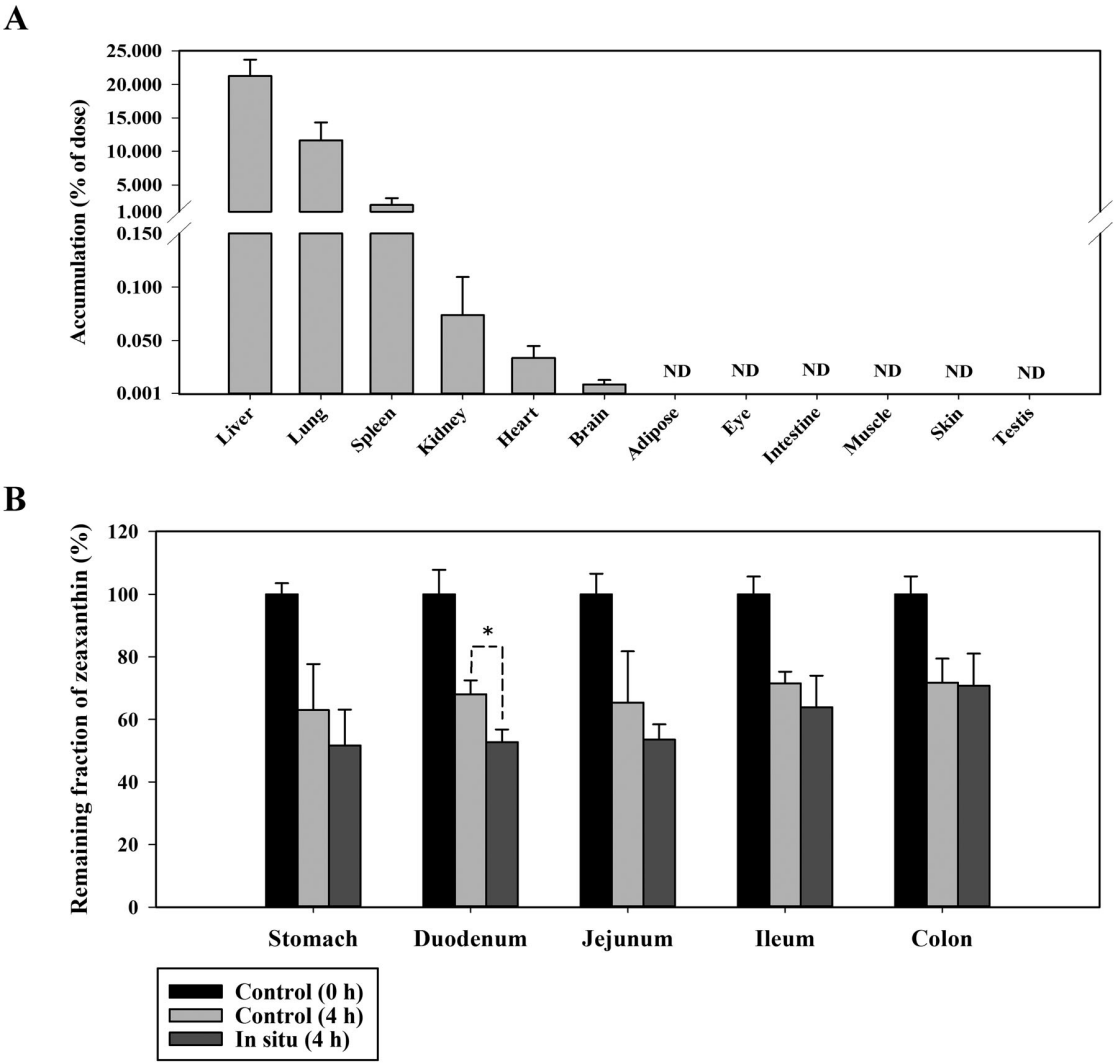
(A) Remaining fractions of zeaxanthin at 4 h after its injection into rat duodenum, jejunum, and ileum loops. (B) Tissue distribution (expressed as % of dose) of zeaxanthin at 90 min after intravenous administration at 5 mg/kg in rats. The rectangular bars and error bars represent the means and standard deviations, respectively (*n* = 5).

Figure 5(A) shows the remaining fractions of zeaxanthin at 4 h after injecting the zeaxanthin suspension into the *in vitro* isolated (control) or *in situ* closed loops of the stomach, duodenum, jejunum, ileum, and colon. It is plausible that both gut luminal degradation and gut absorption of drugs occur simultaneously in the *in situ* closed loops, whereas only gut luminal degradation occurs in the *in vitro* isolated loops. Thus, the differences between the remaining fractions observed in the *in situ* and *in vitro* loops correspond to the actual fractions of drugs absorbed from the loops (Chuesiang et al. 2022). As shown in Figure 5(A), there were no significant differences in the remaining fractions in the stomach, jejunum, ileum, and colon loops between the *in situ* and *in vitro* states (*p* = 0.354 for stomach, 0.299 for jejunum, 0.203 for ileum, and 0.900 for colon). In contrast, the remaining fractions in the duodenum loops differed significantly between the two states (*p* = 0.0119). These results indicate that the gut absorption of zeaxanthin could occur in the duodenum rather than in the other segments; the actual fraction of zeaxanthin absorbed from the duodenum loops can be calculated as 15.3%. Figure 5(B) shows the tissue distribution profiles of zeaxanthin at 90 min following an intravenous dose of 5 mg/kg in rats. Notably, considerable portions of the administered dose (2.0–21.3%) were distributed in the liver, lung, and spleen. In contrast, minimal portions of the dose were found in other tissues (≤0.0987% of the dose). Table 4 shows *in vivo* lymphatic absorption of zeaxanthin after oral administration of four different zeaxanthin suspensions prepared with four different types of the oil phase, i.e., corn oil, stearic acid, oleic acid, and linoleic acid. The blood concentrations of zeaxanthin for 120 min were below the quantitation limit, whereas quantifiable amounts of zeaxanthin were recovered from the liver and lymph tissues. Notably, the zeaxanthin levels in the lymph were significantly higher than those in the liver. Moreover, there were no significant differences in the liver concentrations, lymph concentrations, and lymph‑to‑liver concentration ratios (indicating lymphatic transport efficiency) of zeaxanthin among the test formulations containing four different types of lipid (*p* = 0.124, 0.203, and 0.678, respectively).

## Discussion

The present study aimed to develop an efficient bioanalytical HPLC method for zeaxanthin and elucidate the biopharmaceutical and pharmacokinetic factors that determine the gut absorption and systemic disposition of zeaxanthin in rats. Several bioanalytical conditions, such as light wavelength, stationary/mobile phase, and sample pre-treatment procedure, have been optimized to achieve acceptable chromatographic separation of analytes in biological matrix. The chemical structure of zeaxanthin contains a conjugated polyene chain that acts as a light absorber (Krinsky et al. 2003). As shown in Figure 1, the maximum light absorbance of zeaxanthin occurs at 450 nm, which falls within the range of blue light wavelengths (400–500 nm). Thus, retinal zeaxanthin can reduce blue light exposure to the retina, exerting a protective effect on AMD development (Murillo et al. 2019). The method validation data presented herein indicate that the proposed method is precise, accurate, and reproducible, with a high extraction recovery and minimal matrix effect. Moreover, the method offered good sensitivity (LLOQ of 10 ng/mL), which was more sensitive than previously reported bioanalytical LC‑MS/MS methods (LLOQ of 1406 ng/mL) (Hrvolova et al. 2016).

The plasma pharmacokinetics of zeaxanthin following intravenous administration was linear in the tested dose range. However, the plasma concentrations of zeaxanthin following oral administration were below the quantitation limit, suggesting an extremely low oral bioavailability of zeaxanthin. Oral bioavailability can be calculated as the product of three factors: fraction absorbed from the gut (*F*_abs_), gut availability (*F_G_*), and hepatic availability (*F_H_*). The results depicted in Figure 3 suggest that the gut luminal solubility and stability of zeaxanthin are quite low, which could limit the gut absorption of zeaxanthin *in vivo*. The *F*_abs_ of a drug can be estimated from the *A*_G24h_ values observed after oral dosing, which is valid only when the drug is stable in the gut lumen for 24 h (Han et al. 2021). Thus, this method may be thus inadequate for zeaxanthin that was unstable in SGF and SIF (Figure 3(C)). The absorption of zeaxanthin from the gut tract was further investigated using a closed‑loop study. The results depicted in Figure 5(A) suggest that the gut absorption of zeaxanthin occurs primarily in the duodenum; 15.3% of the zeaxanthin dose was absorbed from the duodenal loop for 4 h. If these data can be extrapolated to *in vivo* conditions, it is speculated that most of the zeaxanthin dose (at least ≥84.7% of the dose) would remain unabsorbed in the gut following oral administration. This implies that gut absorption of zeaxanthin may be limited.

A previous study on lutein, a structural analogue of zeaxanthin, reported that large fractions of the intravenous dose were distributed into the liver, spleen, and lung (in the descending order) (Itagaki et al. 2006). As shown in Figure 5(B), zeaxanthin showed a tissue distribution pattern similar to that of lutein. Thus, the relatively high *V*_ss_ of zeaxanthin (1919–3484 mL/kg; Table 3) could be attributed to its extensive accumulation into the three different tissues. Following intravenous administration, the urinary and gut (including biliary) excretion of zeaxanthin was negligible (Table 3), suggesting that zeaxanthin was eliminated primarily by metabolic processes. CMO2 is mainly responsible for zeaxanthin metabolism in mammals, such as rats and humans (Mein et al. 2011). This enzyme is expressed in various human tissues, including the liver, heart, muscle, and testis (Lindqvist et al. 2005). Moreover, the CMO2 protein is highly expressed in the rat liver (hepatocytes) and intestine (mucosal epithelium) (Raghuvanshi et al. 2015). To examine *in vivo* hepatic metabolism of zeaxanthin, *in vivo* systemic exposure levels of zeaxanthin observed after intraportal administration were compared with those after intravenous administration (Figure 4(B)). The hepatic clearance (CL_H_) of zeaxanthin can be estimated to be 31.2–49.8 mL/kg from the observed *E_H_* of 0.623 and reported hepatic blood flow rate of 50–80 mL/kg (Kim et al. 2021). By comparing the CL_H_ and blood CL (calculated as plasma CL/R_B_) of zeaxanthin, it can be inferred that the liver is the major eliminating organ of zeaxanthin, accounting for 53.5–90.1% of the total clearance process. This indicates minor contributions of extrahepatic organs, including the gut, to the entire process of systemic elimination, which can reconfirm the suitability of the present *in vivo* metabolism study focussing on the liver.

**Table 3.** Noncompartmental plasma pharmacokinetic parameters of zeaxanthin following intravenous (IV) and oral (PO) administration in rats (*n* = 5–6).

IV parameter	5 mg/kg	10 mg/kg
AUC (μg·min/mL)	109 ± 26	205 ± 44
*t*_1/2_ (min)	161 ± 25	169 ± 13
CL (mL/min/kg)	47.7 ± 9.6	50.3 ± 8.9
*V*_ss_ (mL/kg)	3548 ± 1003	3640 ± 768
*A*_U24h_ (% of dose)	BLQ^a^	BLQ^a^
*A*_G24h_ (% of dose)	BLQ^a^	BLQ^a^
PO parameter	20 mg/kg	100 mg/kg
AUC (μg·min/mL)	BLQ^a^	BLQ^a^
*A*_U24h_ (% of dose)	BLQ^a^	BLQ^a^
*A*_G24h_ (% of dose)	28.5 ± 9.0	34.9 ± 12.6

^a^Below limit of quantification.

The absorption of zeaxanthin from the gut lumen into the systemic circulation occurs *via* two different routes: the hepatic portal system and the lymphatic system *via* chylomicron formation (Deming and Erdman 1999; Murillo et al. 2019). In the latter route, a drug absorbed from the gut lumen bypasses hepatic first‑pass elimination. The results shown in Table 4 clearly indicate that the lymphatic pathway plays a significant role in the oral absorption of zeaxanthin. It is also suggested that the lymphatic absorption and bioavailability of zeaxanthin were not affected by the concomitant use of the lipids tested in this study. However, the use of more advanced and sophisticated formulation systems with other lipids and excipients could lead to different results, which warrants further research.

**Table 4.** *In vivo* lymphatic absorption of zeaxanthin after oral administration of zeaxanthin suspension containing four different lipids (*n* = 3).

Formulations	Lipid	Concentration (ng/g tissue)		Lymph/Liver ratio
		Liver	Lymph	
F1	Corn oil	26.1 ± 2.7	186 ± 58*	7.3 ± 3.0
F2	Stearic acid	27.0 ± 3.3	217 ± 84*	8.1 ± 3.2
F3	Oleic acid	36.5 ± 8.2	267 ± 68*	7.3 ± 0.3
F4	Linoleic acid	32.5 ± 4.9	302 ± 37*	9.5 ± 2.5

*Significantly different from the corresponding liver concentration (*p* < 0.05).

This study presented rat pharmacokinetic data obtained after a single intake of synthetic zeaxanthin. As these experimental conditions may not fully reflect actual clinical situations, they need to be further extended to more clinically relevant studies with long‑term multiple intakes of natural products containing zeaxanthin and other various active phytochemicals. Moreover, plasma concentration levels of zeaxanthin after oral administration were below the quantitation limit. Thus, if possible, the sensitivity of the bioanalytical method used in this study needs to be improved to assess reliably the oral pharmacokinetic profiles of zeaxanthin. All the present results taken together, it is suggested that the poor oral bioavailability of zeaxanthin can be attributed primarily to the interplay between low gut luminal solubility/stability, limited gut absorption, and hepatic first‑pass metabolism. Therefore, to enhance the oral bioavailability of zeaxanthin, the vehicle system used for the present study can be further optimized using formulation strategies to modify these factors. For instance, solubility/permeation enhancers and lipid‑based micro/nano‑colloidal systems to enhance solubility/permeability and target lymphatic pathways could serve as feasible tools for efficient oral delivery of zeaxanthin. Furthermore, the information regarding the disposition characteristics of zeaxanthin could be useful for designing formulations administered *via* other routes, such as parenteral, pulmonary, and percutaneous routes.

## Conclusions

Physicochemical compatibility, gut absorption, and systemic disposition of zeaxanthin were comprehensively assessed using relevant rat models. Zeaxanthin exhibited poor solubility and stability in simulated gut luminal fluids. Gut absorption of zeaxanthin occurred primarily in the duodenum, but the major fractions of the dose remained unabsorbed across the entire gut tract. This *in vivo* pharmacokinetic study revealed that the liver is the major organ responsible for the tissue distribution and systemic metabolism of zeaxanthin. Taken together, the poor oral bioavailability of zeaxanthin can be attributed mainly to its poor gut luminal solubility/stability, limited gut absorption, and hepatic first‑pass metabolism. To our knowledge, this is the first systematic study to report the oral absorption and systemic disposition of zeaxanthin from the biopharmaceutical and pharmacokinetic perspectives.
